# Calcium polystyrene sulfonate–induced colitis: advanced characterization of crystal nature with infrared spectroscopy

**DOI:** 10.1093/ckj/sfae210

**Published:** 2024-07-08

**Authors:** Helena Vidal, Vilma Salgado, Patrícia Alves, Nuno Moreira Fonseca1, Vincent Frochot, Aníbal Ferreira

**Affiliations:** Nephrology Department, Local Health Unit of Saint Joseph – Curry Cabral Hospital, Lisbon, Portugal; Nephrology Department, Hospital do Divino Espírito Santo de Ponta Delgada, Azores-São Miguel, Portugal; Lisbon Clinical Academic Center, Lisbon, Portugal; Lisbon Clinical Academic Center, Lisbon, Portugal; Pathology Department, Centro Hospitalar Universitário de Lisboa Central EPE, Lisbon, Portugal; Nephrology Department, Local Health Unit of Saint Joseph – Curry Cabral Hospital, Lisbon, Portugal; Lisbon Clinical Academic Center, Lisbon, Portugal; Nephrology Department, Local Health Unit of Saint Joseph – Curry Cabral Hospital, Lisbon, Portugal; Lisbon Clinical Academic Center, Lisbon, Portugal; Nova Medical School, Nova University of Lisbon, Lisbon, Portugal; Physiology Department, Hôpital Tenon, APHP, Paris, France; Faculty of Medicine, Pierre et Marie Curie University, Paris, France; INSERM UMRS 1155, Paris, France; Nephrology Department, Local Health Unit of Saint Joseph – Curry Cabral Hospital, Lisbon, Portugal; Lisbon Clinical Academic Center, Lisbon, Portugal; Nova Medical School, Nova University of Lisbon, Lisbon, Portugal

**Keywords:** calcium polystyrene sulfonate, colonic injury, diarrhea, hyperkalemia, infrared spectroscopy

## Abstract

Classical potassium binders are used in the treatment of hyperkalemia and are widely associated with gastrointestinal side effects, with crystal colonic injury being rare but potentially fatal. In this report, we describe the case of an 82-year-old male with hyperkalemia and calcium polystyrene sulfonate crystal–associated colonic necrosis. Traditionally, this diagnosis has relied on the examination of crystal morphology and polarization through microscopy. Our study enhances crystal identification by incorporating an analysis of the physical characteristics of the crystals using infrared spectroscopy. This is the first description, to our knowledge, of the calcium polystyrene sulfonate infrared spectrum.

## BACKGROUND

Hyperkalemia, characterized by a serum potassium level exceeding 5.5 mEq/L, is a prevalent clinical problem primarily arising from compromised urinary potassium excretion due to acute or chronic kidney disease. This electrolyte imbalance leads to numerous consequences, including muscle weakness, paralysis and potentially fatal cardiac arrhythmias [1]. Classical potassium binders (CPB), such as sodium polystyrene sulfonate (SPS) and calcium polystyrene sulfonate (CPS), have been used to treat this condition since 1958 [2]. Their mechanism of action involves the exchange of potassium for sodium or calcium, respectively, primarily in the gastrointestinal tract, especially the colon. However, CPB have been associated with various gastrointestinal side effects such as nausea, vomiting, bowel obstruction and diarrhea. The first case of colonic injury attributed to polystyrene sulfonate was documented in 1987 by Lillemoe *et al.* [3]. CPB can elicit epithelial damage via osmotic action and induce ischemic lesions through vasospasm of the intestinal vessels. Histological examination unveils non-polarizable CPB crystals with a rhomboid shape, mosaic or “fish scale” pattern exhibiting basophilic and dark purple characteristics, and positive staining with periodic acid–Schiff [4].

## CASE REPORT

The case is a hospitalized 82-year-old male, with a medical history notable for stage 3b chronic kidney disease of undetermined etiology, untreated hypertension and a prior ischemic stroke. His only medication is alprazolam, at a dosage of 2.5 mg per day. He presented with muscle weakness, nonspecific malaise and anorexia. Physical examination was significant for hypotension (94/55 mmHg) and dehydrated mucous membranes. Laboratory workup revealed acute on chronic kidney disease with serum creatinine levels rising from 2.2 mg/dL (for 7 years and since then without further analytical control or medical follow-up) to 8.7 mg/dL [reference range (RR) between 0.67 and 1.17 mg/dL], urea from 79 mg/dL to 231 mg/dL (RR 16.6–48.5 mg/dL) and potassium from 5 mEq/L (RR 3.5–5.5 mEq/L) to 5.9 mEq/L. The electrocardiogram was normal. An abdominal computed tomography scan revealed marked bilateral renal atrophy, bilateral hydronephrosis without obstruction evidence and notably thickened bladder walls, prompting the insertion of a urinary catheter.

The patient was admitted to the medical ward and started on intravenous fluids (sodium chloride 0.9%) and CPS at a prescribed dose of 20 g twice daily, along with a potassium-restricted diet. Subsequent to admission, ertapenem was initiated for the treatment of a urinary tract infection, while amlodipine was administered to manage hypertension. A comprehensive therapeutic evaluation was undertaken, with particular attention given to medications associated with the potential for hyperkalemia, notably renin–angiotensin system inhibitors, which were subsequently excluded. Arterial blood gas analysis revealed metabolic acidosis—pH of 7.25 (RR 7.35–7.45) and a bicarbonate level of 15.3 mmol/L (RR 22–26 mmol/L). Oral bicarbonate supplementation was initiated at a daily dose of 1 g. Nevertheless, hyperkalemia persisted at 5.7 mEq/L despite acidemia correction and CPS administration.

Urinary catheterization was performed, yet due to persistent bilateral hydronephrosis and unimproved kidney function (with creatinine levels at 6.2 mg/dL and urea at 213 mg/dL), bilateral ureteral stent placement was required on the 23rd day of hospitalization. At this point the patient developed diarrhea, abdominal pain and bloating, prompting an abdominal computed tomography angiography revealing small intestine and colon distension, along with diffuse thickening of the sigmoid colon. Screening for *Salmonella, Shigella, Campylobacter* and *Clostridium difficile* all returned negative results. There was no elevation in inflammatory parameters; C-reactive protein was at 4.3 mg/L (RR <5.0 mg/L).

**Figure 1:**
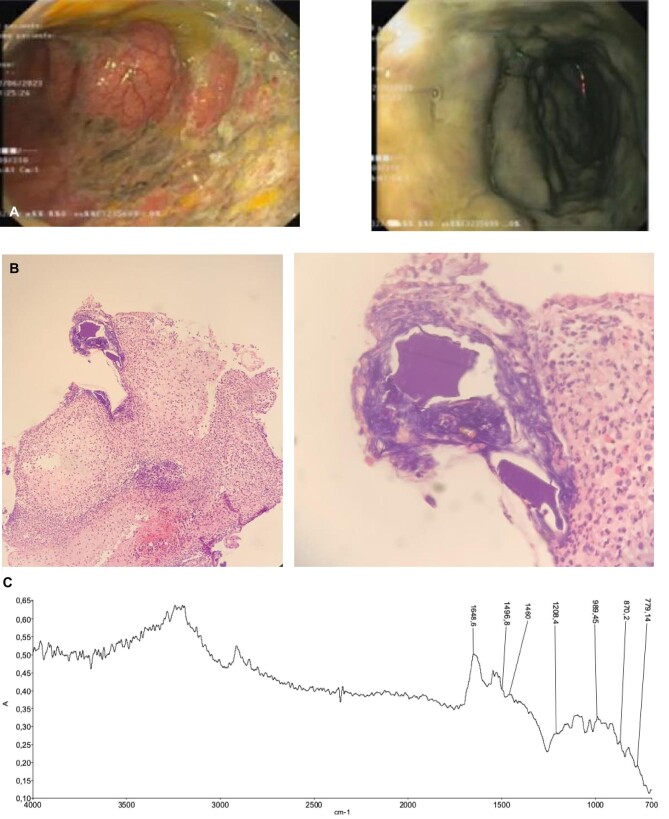
(**A**) Ulcerated mucosa with purplish/grayish areas; (**B**) optical microscopy with hematoxylin and eosin staining revealed inflammatory lesions containing basophilic and purple polygonal crystals in the colonic ulceration, resembling a fish scale pattern; (**C**) Fourier-transform infrared spectrum of crystal deposited in colonic tissue, confirmed to be of CPS crystal origin.

Subsequent to enduring persistent diarrhea and the emergence of hematochezia, a colonoscopy was performed, revealing ulcerated mucosa with purplish/grayish areas (Fig. 1A). On histological examination the ulcerated lesion of the colon consisted of the usual fibrous bed with granulation tissue and a surface of necrotic debris intermingled with numerous basophilic rhomboid crystals with a fish scale–like pattern, suggestive of CPS (Fig. 1B). Considering the histological findings, culture tests and radiological examinations, we have excluded the diagnosis of cancer, inflammatory bowel disease, microscopic colitis, ischemic colitis, *Clostridioides difficile* infection and infectious colitis. The diagnosis of CPS-induced colitis was corroborated through the confirmation that the visually observed crystals were indeed composed of CPS, as demonstrated by infrared spectroscopy (Fig. 1C). Following discontinuation of CPS, the patient experienced diarrhea resolution. However, a recurrence of hyperkalemia (with a value of 6 mEq/L) prompted the commencement of daily patiromer at a dosage of 8.4 g. While potassium levels were effectively managed until the 53rd day of hospitalization, hemodialysis was commenced at this point due to the onset of uremic symptoms and the persistent presence of pronounced azotemia, with creatinine at 5.88 mg/dL and urea at 185 mg/dL.

## DISCUSSION

The use of CPB to treat hyperkalemia traces its origins back to the 1960s. Despite prevalent gastrointestinal side effects, CPB remains extensively employed in contemporary clinical practice. Colonic injury associated with CPB is characterized by gastrointestinal features including diarrhea, bleeding, ischemic colitis, focal and deep ulceration, necrosis, and perforation, with pathological analysis revealing basophilic crystals exhibiting a “fish scale” pattern morphology.

The precise pathophysiological mechanisms of CPB-induced colitis remains uncertain, with suspected contributors including alterations in intestinal microcirculation and the release of prostaglandins. Identifiable risk factors encompass chronic kidney disease, hypotension, hypovolemia, immunosuppression, postoperative status and obstructive bowel disease [5].

Achieving a definitive diagnosis requires unequivocal evidence of the molecular nature of the crystal. While polarized light microscopy and morphological examination are currently prevalent tools in histologic investigations, they prove insufficient for determining the crystal's molecular composition. Notably, there is a potential for confusion, as in the case of cholestyramine crystals being misidentified as CPB crystals. Accurate identification of the crystal's nature requires an analysis of its chemical composition through Fourier-transformed infrared spectroscopy. We have confirmed that the crystal observed in the colon biopsy is indeed CPS by comparing its infrared spectrum with that of CPS powder administered to the patient [FT-IR hyperspectral images were recorded with a Spectrum spotlight 400 FT-IR imaging system (Perkin Elmer, Waltham, MA, USA), with a spatial resolution of 6.25 μm and a spectral resolution of 8/cm].

We have previously applied this technique for the diagnosis of SPS-induced colitis. To the best of our knowledge, this represents the first case of CPS confirmed using this methodology [6].

Hyperkalemia is a common and serious problem in chronic kidney disease that demands strict management through potassium-restricted diets. In refractory cases, the use of new potassium binders such as patiromer, known for improved gastrointestinal tolerance, or sodium zirconium cyclosilicate, with its rapid onset of action, may demonstrate reduction of colitis complications.
